# Insights into the C-Cl Bond Breaking in Epichlorohydrin Induced by Low Energy (<10 eV) Electrons

**DOI:** 10.3390/molecules29246051

**Published:** 2024-12-23

**Authors:** Hassan Abdoul-Carime, Louisa Castel, Franck Rabilloud

**Affiliations:** 1 Universite de Lyon, Université Lyon 1, Institut de Physique des 2 Infinis, CNRS/IN2P3, UMR5822, F-69100 Villeurbanne, France; louisa.castel@ens-lyon.fr; 2 Universite Claude Bernard Lyon 1, CNRS, Institut Lumière Matière, UMR5306, F-69100 Villeurbanne, France; franck.rabilloud@univ-lyon1.fr

**Keywords:** epichlorohydrine, C-Cl bond breaking, low energy (<10 eV) electrons, dissociative electron attachment, DFT calculations

## Abstract

Epichlorohydrin is used as an intermediate for the synthesis of polymers and, more particularly, epoxy adhesives. The traditional process involves the cleavage of the carbon-chlorine bond in an alkaline solution. Here, we investigate the breakage of this bond induced by low-energy (<10 eV) electrons. We show the production of the chlorine anion via a resonant process at different energies. The experimental observations are completed by quantum chemistry calculations of the involved molecular orbitals in the formation of the precursor temporary anions, and their decay mechanisms are discussed in terms of the complex potential energy curve crossing network. The gained information may potentially contribute to a strategy of synthesis by other means where low-energy electrons are implicated, i.e., cold plasmas or even scanning tunnelling microscope for which the bond breakage can be controlled by the energy of the colliding electrons.

## 1. Introduction

Halogen bonds play a pivotal role in chemical synthesis [1] with applications in various fields, such as medicinal drug design [2,3] and agricultural pesticides [4]. In polymer science, the halogenated compound is not widely utilized yet, although recent years have seen an increase in interest in the use of halogen bonding in processes for polymer assembly [5,6,7]. For instance, epoxy adhesives have been shown to be synthesized using epichlorohydrin (ECH, CH_2_CHOCH_2_Cl) as an intermediate in traditional solution processes [8] or via the association of bio-compatible compounds [9,10,11]. In addition to the standard methods for the chemical synthesis of polymers, more sustainable and eco-friendly techniques have been developed, among which are cold (or non-thermal) plasma [12,13,14] or radiation-based processes [15,16,17]. In these techniques, neutral radicals are considered to be responsible for inducing the polymerization reactions. However, the produced secondary electrons are very likely to be implicated.

In either the plasma [18] or radiation [19] techniques, the ballistic electrons are produced abundantly with an energy distribution below 20 eV [20,21]. The ability of these particles to trigger chemical reactions leading to the synthesis of new species is now demonstrated. For instance, it has been shown that the irradiation of a film of dimethyl sulfide with electrons of energies as low as 1 eV produces ethylene via the decomposition of the precursor molecules via resonant processes [22]. These electrons are also capable of synthesizing polymers. Indeed, the irradiation of films of methanol (CH_3_OH) by a 20 eV electron beam generates ethylene glycol (HOCH_2_CH_2_OH) [23]. At these energies, the interacting electrons may ionize, excite, or fragment the molecule as the first step, followed by the reactions with the environing molecules. Therefore, it is important to understand this primary action and, more particularly, the fragmentation at low energies. For the halogenated compounds, the molecular decomposition evolving the C-Cl cleavage is particularly efficient at energies usually near 0 eV. The cross-sections for such a bond rupture induced by electrons, which quantifies the efficiency of the process, have been estimated to be as high as 10^−13^ cm^2^ (from tetrachlorocarbon [24]) or 10^−14^ cm^2^ (chlorouracil) [25], respectively. Thus, it is desirable to investigate the interaction of electrons with the halogenated molecules in isolation in the gas phase by exploring and quantifying the accessible fragmentation channels prior to potentially applying the compounds for synthesis using techniques that involve electrons.

In this work, we study the collision of low-energy electrons with epichlorohydrin in the gas phase and particularly the process underlying the cleavage of the carbon–chloride, C-Cl, bond. We show that the fragmentation of epichlorohydrin produces mainly the Cl^−^ anion at the electron energies above 1.5 eV via resonant processes, supported by DFT calculations.

## 2. Results and Discussion

**Figure 1:**
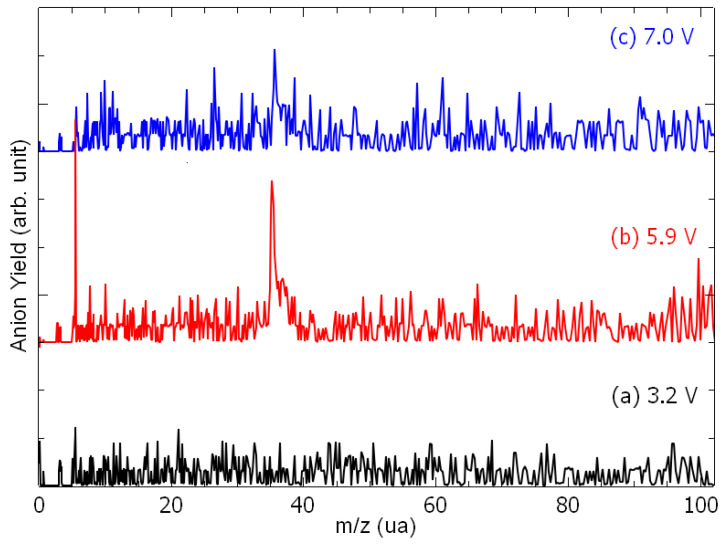
Anion time-of-flight mass spectra recorded at the electron accelerating voltage of (**a**) 3.2 V, (**b**) 5.9 V, and (**c**) 7.0 V.

**Figure 2:**
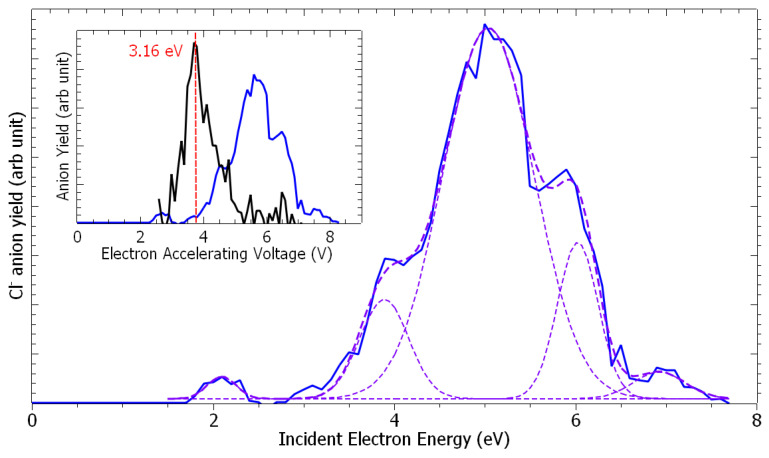
Cl^−^ anion yield as the function of the electron energy. In the inset, the yield of (ethylenediamine-H)^−^ and Cl^−^ anions (black and blue lines, respectively) as the function of the electron accelerating voltage. The dashed lines are guide-to-the-eyes.

Figure 1 presents the anion time-of-flight spectra recorded from the collision of electrons with epichlorohydrin molecules, recorded at different electron accelerating voltages (EAV). It is to be noted here that the residential time of the formed ion species in the extraction and the acceleration areas are estimated to be 250 ns and 150 ns, respectively. If these time constraints are verified, they will arrive as a peak at the appropriate time in the time-of-flight spectra shown in Figure 1. At an EAV of 3.2 V, no anion is detected (Figure 1a). As the EAV increases (Figure 1b,c), a peak is observed at *m*/*z* 35 with an additional feature at *m*/*z* 37 (Figure 1b). These species are assigned to the ^35^Cl^−^ and ^37^Cl^−^ negative fragments. No further anion is detected. At each EAV, the chlorine yield is integrated and normalized to the electron current. Figure 2 shows the Cl^−^ anion yield as the function of the electron energy. The conversion from the electron accelerating voltage to the electron energy is obtained by shifting the EAV value by using a calibrating molecule [26], i.e., ethylenediamine (EDA) is used in this work [27]. The inset of Figure 2 presents the yield function of the dehydrogenated ethylenediamine anion (black line), for which the resonance peak has been estimated to be 3.16 eV [27], while the blue line corresponds to that of the chlorine anion. Thus, the Cl^−^ anion yield function exhibits structures peaked at 2.2 eV, 3.9 eV, 5.1 eV, 6.1 eV, and 6.9 eV (Figure 2). It is to be noted at this point that the appearance energy of the chlorine anion is observed at well above 1.5 eV, while the cleavage of the C-Cl bond yielding the chlorine anion is nearly a thermoneutral reaction when considering the electron affinity of the chlorine atom (c.a., 3.6–3.7 eV [28]) and the average C-Cl bond dissociation energy (c.a., 3.65 eV [29]).

**Figure 3:**
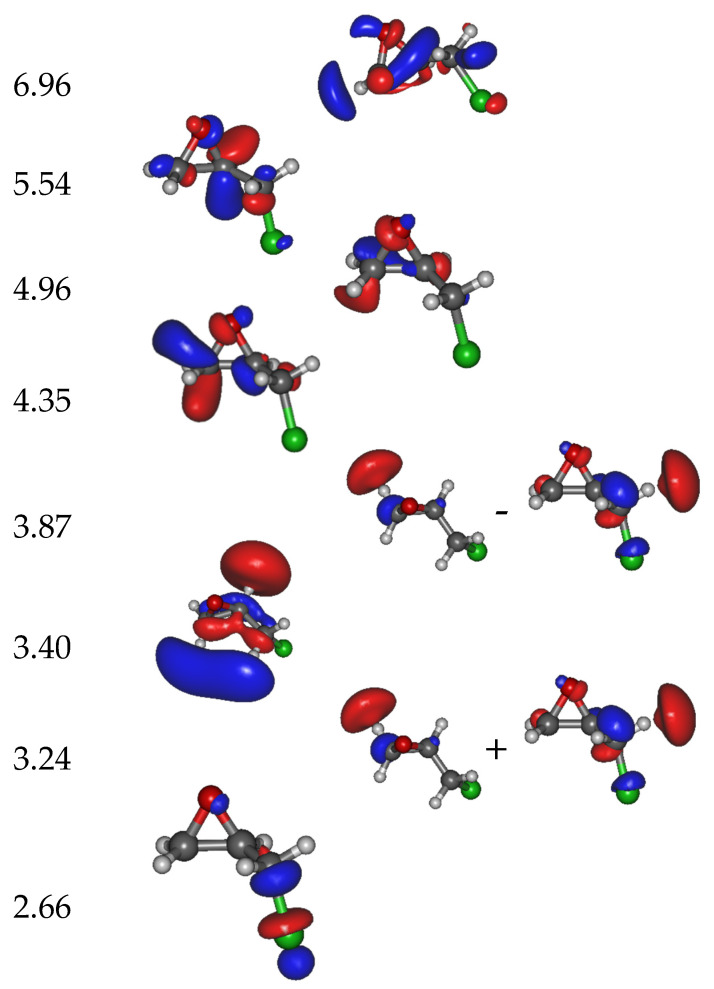
Calculated electron attachment energies (in eV) together with the main molecular orbitals involved in the trapping of the extra electron. The state located at 2.66 eV possesses a $\sigma_{C-Cl}^{*}$ bond character; those at 3.24, 3.40, and 3.87 eV have $\sigma_{C-H}^{*}$ bonds, while states at higher energy show delocalized orbitals with a $\sigma_{C-O}^{*}$ bond. The white, grey and green balls represent H, C and Cl atoms, respectively.

**Figure 4:**
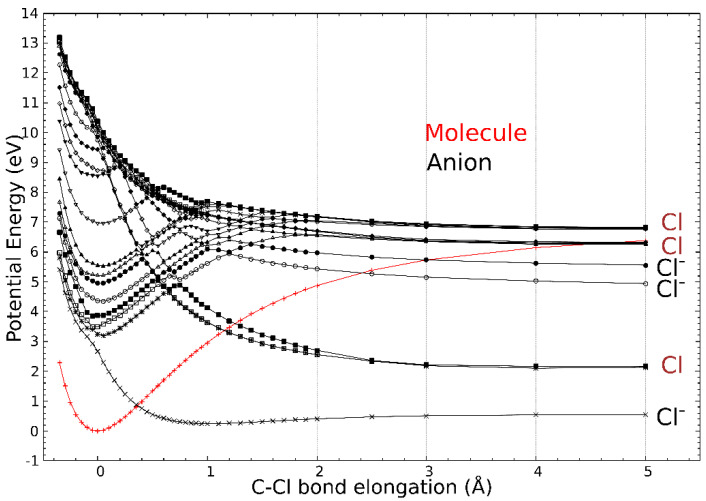
Potential energy curves as the function of the C-Cl bond elongation (Å). The equilibrium distance of the molecule is taken as the reference. The neutral molecule PEC is plotted in red, while the anion PECs are in black. The charge state of the dissociated chlorine atom/anion at the asymptote is given as Cl or Cl^−^.

The calculated molecular orbitals (MOs) associated with the different anion states are displayed in Figure 3. The first state (corresponding to the trapping of the electron in the Lowest Unoccupied Molecular Orbital (LUMO)) is located at 2.66 eV above the neutral ground state. In the next seven states, i.e., with the resonance electron attachment energies at 3.24, 3.40, 3.87, 4.35, 4.96, 5.54, and 6.96 eV, the excess electron resides mainly at the (ECH-Cl) moiety of the molecule. The 3.24 eV and 3.87 eV states are formed by a linear combination of two main MOs. Finally, the potential energy curves (PECs) of the neutral and the anion as the function of the C-Cl distance are provided in Figure 4. The curve of the first anionic state (calculated at 2.66 eV in a vertical attachment process) decreases to an asymptote, corresponding to the formation of a chlorine ion. The presence of several avoided crossings from the lowest energy states around a zero Å elongation is to be noted.

The peaks observed in the chlorine anion yield function (Figure 2) indicate that the dissociation of the C-Cl bond is controlled by resonant processes. At these energies below the first valence ionization energy of the molecule, c.a., 10.64 eV for ECH [30], it is now well-admitted that dissociative electron attachment (DEA) is the most efficient fragmentation process [31]. In brief, the colliding electron is temporarily trapped by the precursor to form a transitory negative ion; this latter may undergo dissociation into a negative fragment (Cl^−^) and a neutral counterpart (ECH-Cl)^•^, which is, in this case, a neutral radical. The DEA cross-section, reflecting the production of the anions, is a convolution of the electron capture cross-section with the survival probability (i.e., the autodetachment vs. dissociation times) [31]. The capture of the extra electron may arise from the shape or core-excite resonances [31] but also possibly via the formation of a dipole-bound anion as the gateway for DEA [32]. For this latter process, the dipole moment of the molecule must be sufficiently high, i.e., above 2 D [33], to sustain a dipole-bound state. The low dipole moment value of ECH calculated here of 0.5627 D forbids such a state to be formed, thus prohibiting molecular dissociation via this mechanism. The shape resonance consists of the accommodation of the colliding electron into an empty MO. For the core-excited resonance process, one of the valence electrons is excited while the extra-electron is concomitantly trapped by the positive molecular core. The 2.2 eV energy peak position observed in the Cl^−^ anion yield function (Figure 2) arises from the shape resonance via the occupation of the extra electron in the state at 2.66 eV, possessing a C-Cl bond with a σ * character, shown in Figure 3. The local optimization of the anion, shown in Figure S2, exhibits a different geometry, which involves a wagging of the CH_2_ group and the shift of the chlorine atom: the energy is lowered by 2.92 eV, and so the optimized anion lies 0.26 eV below the neutral. The state at 2.66 eV is purely dissociative, as shown by the lowest anion PEC that presents, however, a shallow minimum (Figure 4, crosses). While the MO is calculated at 2.66 eV, the difference with the observed peak position (2.2 eV) in the Cl^−^ anion yield function may result in the convolution of the capture cross-section and the survival probability of the transitory precursor anion. The next MOs, calculated at 3.24 eV, 3.40 eV, and 3.87 eV, show that the excess electron is not mainly residing on the chlorine moiety and, if dissociation arises, the (ECH-Cl)^−^ anion fragment would be formed along with the Cl^•^ radical. Moreover, the PECs curves (full and open squares and stars) associated with these MOs present a potential barrier of more than 1.0 eV to overcome for such a dissociation to arise. The experimental measurements show neither the parent (ECH)^−^ nor (ECH-Cl)^−^ since no clear peaks are observed in the anion mass spectra at *m*/*z* 92 and 57, respectively, indicating that these dissociation channels are not likely or at least less probable than that for production of the chlorine anion. It is to be noted that the electron affinity of (ECH-Cl)^•^, calculated to be 0.18 eV after the relaxation of the geometry of the radical, is much lower than that of the Cl^•^ radical mentioned above [27]. A resonant peak is observed at 3.9 eV in the Cl^−^ yield function, which may correspond to the trapping of the electron in the state calculated at 3.87 eV. The dissociation channel for the Cl^−^ anion may then operate via the crossing of the PECs (full squares ⟶ crosses) at the ECH-Cl bond elongation of 0 Å (i.e., the equilibrium position of the neutral). The intense observed 5.1 eV peak may be associated with the calculated states above 5 eV, which can lead to fragmentation into Cl^−^ either via the coupling of the PECs through the lowest pathway or by opening a new dissociation pathway leading to the excited state of the anion Cl^−^ (asymptote at ~5 eV in Figure 4). It is to be noted that the PECs are calculated at 0 K. The height of the potential barriers calculated at this temperature is likely to be reduced (typically by at least 0.5 eV) at the experimental conditions (T~300 K), rendering further dissociation pathways accessible.

From the inset of Figure 2, the comparison of the (EDA-H)^−^ anion yield with that of the chlorine anion can provide an estimation of the Cl^−^ anion production cross-section. Indeed, the anion production cross-section, σ_ion_(E), at a given incident electron energy, E, can be estimated via N_ion_(E) = ε·N_e_·N_neutra_·σ_ion_(E)·L, where N_ion_ represents the number of collected ions, ε represents the detection efficiency, N_e_ and N_neutral_ represent the number of colliding electrons and the density of the neutral target molecules, respectively, and L represents the length of the interaction region. Since N_neutral_ and L are not accessible, σ_Cl_^−^/σ_(EDA-H)_^−^ can be obtained by knowing the gas pressure ratio, P_ECH_/P_EDA_ (i.e., ~2, in the present work), and the measured numbers of ions N_Cl_^−^/N_(EDA-H)_^−^. From the yield functions of the Cl^−^ and (EDA-H)^−^ anions (blue and black lines, respectively, in the inset of Figure 2) and with the known σ_(EDA-H)_^−^ at 3.16 eV (c.a., 1.4 × 10^−15^ cm^2^ [27]), the cross-section for the production of the chlorine anion can be estimated at the electron energy of 2.66, 3.9, 5.1, 6.1, and 6.9 eV to be 1.2 × 10^−16^ cm^2^, 3.4 × 10^−16^ cm^2^, 1.7 × 10^−15^ cm^2^, 7.3 × 10^−16^ cm, and 1.2 × 10^−16^ cm^2^, respectively.

## 3. Methodologies

### 3.1. Experimental Method

The experimental setup has been thoroughly described elsewhere [26,34]. Only the essentials of the method will be provided here. The cross-beam experiment, working at ultra-high vacuum conditions (base pressure of 6 × 10^−9^ mbar), is composed of a molecular beam, a double counter-propagating electron beams produced by two electron guns (EG1 and EG2), and a dual (+/−) time-of-flight mass spectrometer (TOF_MS), all mounted orthogonally. The epichlorohydrin (Sigma-Aldrich, Saint-Quentin-Fallavier, France, 99%) is injected perpendicularly to the electron guns and TOF_MS plane, leading to the increase in the chamber pressure to 1.7-to-2.0 × 10^−6^ mbar. EG1, equipped with a trochoidal monochromator based on a dispersive **ExB** field, provides a mono-kinetic electron beam of few nA with a typical energy resolution of 300 meV. EG1 is used for inducing the electron–molecule collision experiments. The role of the EG2 is to ionize the parent molecule or fragment formed after the fragmentation of the precursor. A dual (+/−) time-of-flight mass spectrometer (TOF-MS) detects the negative and ionized neutral species that are produced after the collision of the target molecule with electrons from EG1 or/and EG2. The negative and positive ions are expelled from the collision area by a −450 V, 600 ns pulse, and they are accelerated by +1450 V and −200 V, respectively (acceleration area), before reaching the free field zone for the time separation. They are collected by a pair of multi-channel plates (MCPs), transforming the arriving ions into electric pulses. These are converted by a Time-Digital-Converter to arrival times and are stored in a PC for the ‘off line’ analysis. Note that with this experimental arrangement, it is possible to systematically verify the molecular beam (e.g., purity of the investigated product) prior to further electron collision studies.

### 3.2. Theoretical Method

Calculations have been performed in the framework of the density-functional theory (DFT) and the time-dependent DFT (TDDFT) using the Gaussian16 suite of programs [35]. The exchange and correlation potential is that of the range-separated hybrid density functional ωB97xd [36]. The resonance electron attachment energies are calculated following the methodology presented in Ref. [37]; the ground state is calculated using an extended basis set aug-cc-pvtz, while the anionic excited states are obtained using the non-augmented basis set cc-pvtz [38]. This computational level has been previously shown to be adequate to describe valence anionic excited states with good accuracy while preventing the occurrences of intruding discretized continuum states in the excited state spectra method [37].

Epichlorohydrin presents three stable conformers (Figure S1), which differ by the C-C-C-Cl dihedral angle [39]. Only the most stable structure is considered in the present study. Pre- and post-processing operations are performed by using the graphical interface Gabedit [40].

## 4. Conclusions

The dissociation of epichlorohydrin induced by low energy (<10 eV) electrons produces chlorine anion associated with the (ECH-Cl)^•^ neutral radical. Multi-body fragmentation, particularly at high energies, i.e., Cl^−^ anion associated with more neutral fragments, was not clearly observed by using the ionization gun, EG2. The resonance states involved in the dissociation of the transitory anion are identified and the dissociation pathways can be suggested from the potential energy curve crossing network.

The information gained by this study possibly contributes to control synthesis since electron energy controls the breaking of specific breaking of chemical bonds. Such a selective chemistry has already been shown previously for the benzonitrile/water system. Indeed, irradiating the admixture by a 3–4 eV electron beam only damages benzonitrile via the C_6_H_5_-CN bond cleavage, yielding CN^−^ anion and reactive phenyl radical [41] while leaving H_2_O unaffected [42]. The further reaction of the phenyl radical with water molecule synthetizes phenol [43]. In the present case, the possible reaction of the admixture of C_6_H_5_CN:CH_2_CHOCH_2_Cl, triggered by a 4 eV electron beam for the synthesis of C_6_H_5_-CH_2_CHOCH_2_ via the reaction C_6_H_5_^•^ and (ECH-Cl)^•^ radicals is currently under investigations. Such a molecule has not been reported in the literature yet and, thus, if produced, could open access to some new perspectives for selective synthesis methods based on the fundamentals of the low-energy electron–molecule interaction.

## Data Availability

Data are contained within the article and Supplementary Materials.

## Supplementary Materials

The following supporting information can be downloaded at: https://www.mdpi.com/article/10.3390/molecules29246051/s1. Figure S1: Optimized configurations. Figure S2: (a) Local optimization of the anion, (b) density of the extra electron, (c) energy diagram.
