# Supplementary material for: Insights into the C-Cl Bond Breaking in Epichlorohydrin Induced by Low Energy (<10 eV) Electrons
Source: Molecules. 2024 Dec 23;29(24):6051. doi: 10.3390/molecules29246051 (PMC11677569; doi:10.3390/molecules29246051)

Insights into the C-Cl Bond Breaking in Epichlorohydrin Induced by Low Energy (<10 eV) Electrons

Hassan Abdoul-Carime [1,*], Louisa Castel [1] and Franck Rabilloud [2]

[1] Universite de Lyon, Université Lyon 1, Institut de Physique des 2 Infinis, CNRS/IN2P3, UMR5822, F-69100 Villeurbanne, France; louisa.castel@ens-lyon.fr

[2] Universite Claude Bernard Lyon 1, CNRS, Institut Lumière Matière, UMR5306, F-69100 Villeurbanne, France; franck.rabilloud@univ-lyon1.fr

* Correspondence: hcarime@ipnl.in2p3.fr

Figure S1: Optimized configurations.

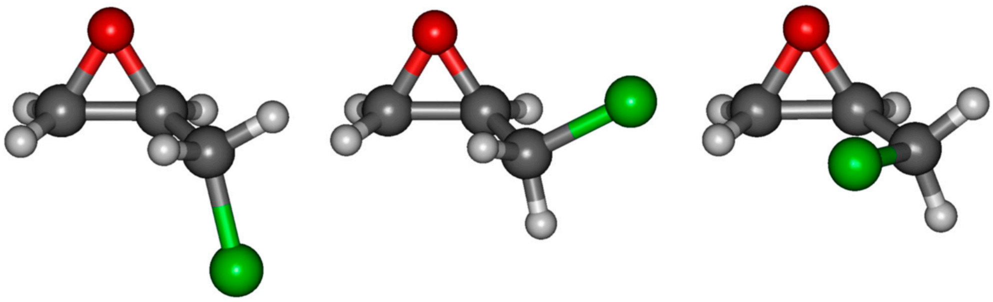

	geo 1 (gauche-II)	geo 2 (gauche-I)	geo 3 (cis)
Relative energy	0.0	+0.02 eV	+0.06 eV

Figure S2: (a) Local optimization of the anion, (b) density of the extra electron, (c) energy diagram.

(a)

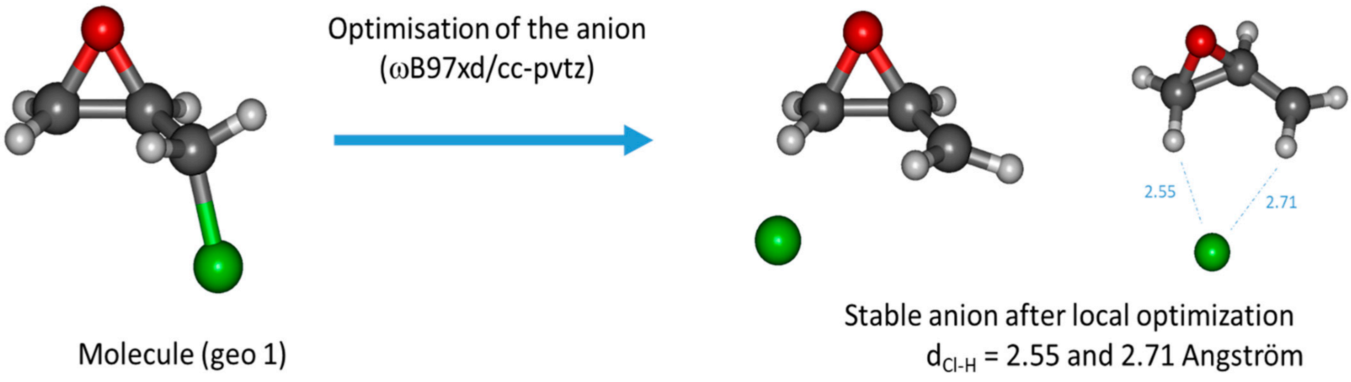

(b)

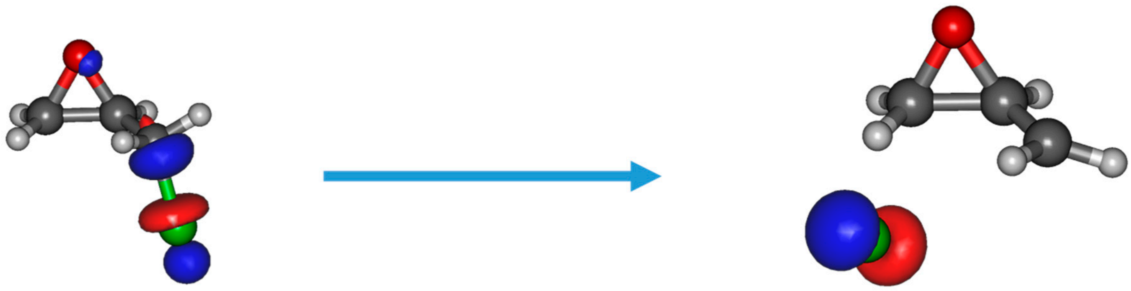

(c)

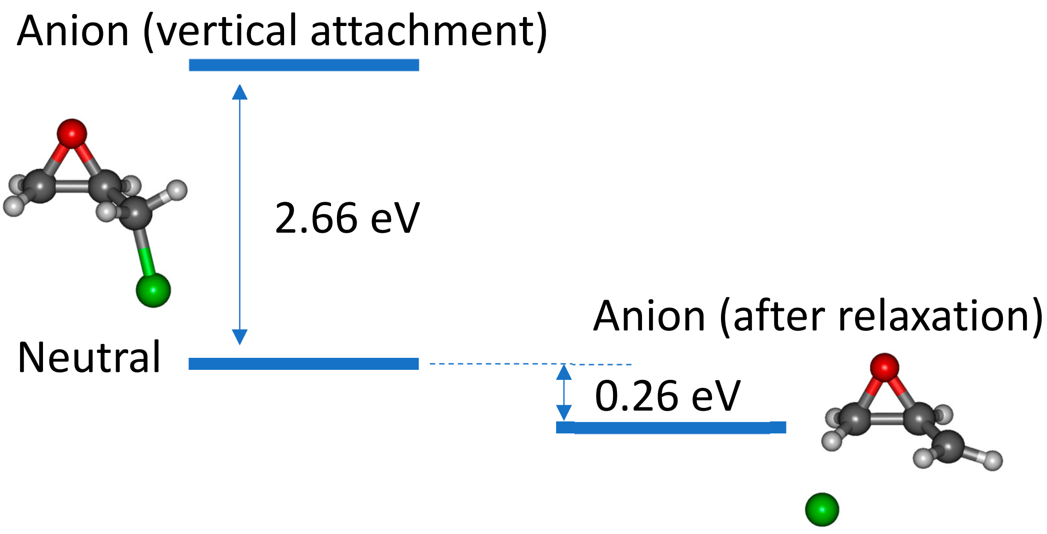

Supplement: Supplementary file 1 [file molecules-29-06051-s001.zip › molecules-3371531-supplementary.pdf]
